# High Oxygen Sensitivity of TiO_2_ Thin Films Deposited by ALD

**DOI:** 10.3390/mi14101875

**Published:** 2023-09-29

**Authors:** Aleksei V. Almaev, Nikita N. Yakovlev, Dmitry A. Almaev, Maksim G. Verkholetov, Grigory A. Rudakov, Kristina I. Litvinova

**Affiliations:** 1 Research and Development Center for Advanced Technologies in Microelectronics, National Research Tomsk State University, 634050 Tomsk, Russia; nik_mr_x@mail.ru (N.N.Y.); almaev001@mail.ru (D.A.A.); verkhmaks@yandex.ru (M.G.V.); 2 Fokon LLC, 248035 Kaluga, Russia; 3 Institute of Nanotechnology of Microelectronics of the Russian Academy of Sciences, 119991 Moscow, Russia; rudakov.g@inme-ras.ru (G.A.R.); litvinova@misis.ru (K.I.L.)

**Keywords:** TiO_2_ films, atomic layer deposition, gas-sensitive properties, oxygen sensors, sensory effect

## Abstract

The gas sensitivity and structural properties of TiO_2_ thin films deposited by plasma-enhanced atomic layer deposition (ALD) were examined in detail. The TiO_2_ thin films are deposited using Tetrakis(dimethylamido)titanium(IV) and oxygen plasma at 300 °C on SiO_2_ substrates followed by annealing at temperatures of 800 °C. Gas sensitivity under exposure to O_2_ within the temperature range from 30 °C to 700 °C was studied. The ALD-deposited TiO_2_ thin films demonstrated high responses to O_2_ in the dynamic range from 0.1 to 100 vol. % and low concentrations of H_2_, NO_2_. The ALD deposition allowed the enhancement of sensitivity of TiO_2_ thin films to gases. The greatest response of TiO_2_ thin films to O_2_ was observed at a temperature of 500 °C and was 41.5 arb. un. under exposure to 10 vol. % of O_2_. The responses of TiO_2_ thin films to 0.1 vol. % of H_2_ and 7 × 10^–4^ vol. % of NO_2_ at a temperature of 500 °C were 10.49 arb. un. and 10.79 arb. un., correspondingly. The resistance of the films increased due to the chemisorption of oxygen molecules on their surface that decreased the thickness of the conduction channel between the metal contacts. It was suggested that there are two types of adsorption centers on the TiO_2_ thin films surface: oxygen is chemisorbed in the form of O^2–^ on the first one and O^–^ on the second one.

## 1. Introduction

Oxygen is the most important gas for human life, and there is a widespread demand for O_2_ sensors and for measuring the O_2_ concentration in ambient environment. Oxygen detection at an over 1 vol. % level with a high accuracy is essential to control the reactive chemical concentration in the chemical industry and metallurgy [1] and to analyze exhaust gas composition of automobile engines [2,3].

TiO_2_ belongs to the large material class of metal oxide semiconductors [4,5,6,7,8,9,10]. It is attractive for developing O_2_ sensors due to its low cost and high chemical and thermal stability [11,12,13,14,15,16,17,18,19,20,21]. Now, commercial O_2_ sensors are based on bulk and thick-film TiO_2_ structures [6]. Such structures are not highly sensitive to O_2_. A well-known method for optimizing gas-sensitive properties of materials is the use of thin film structures [16]. Film thickness *d* plays a key role in the gas sensitivity of thin film structures. It has been shown that the optimal thickness of TiO_2_ films providing a high gas sensitivity should be comparable to the Debye length *L*_D_. The *L*_D_ = 10–50 nm for TiO_2_ at an electron concentration of *n* = 10^16^–10^18^ cm^−3^ and a permittivity of ε_0_ = 18.9. Atomic layer deposition (ALD) is a highly promising method for growing very thin TiO_2_ films (*d* = 10–50 nm) with large homogeneity and reproducibility of structural and electrical properties. The ALD allows deposition of continuous films with high precision control over thickness and impurities levels [21,22].

Previously, the ALD method was used to produce thin films structures of various metal oxide semiconductors, mainly based on SnO_2_ (see Table 1). At the same time, the gas-sensitive properties of such structures were studied under the exposure to low concentrations of toxic gases and H_2_. Detailed studies on the O_2_ sensitivity of ALD-deposited metal oxide films have not been practically carried out. Therefore, the purpose of this work is to gain a deep insight into the gas-sensitive properties of the ALD-TiO_2_ thin films under O_2_ exposure and to explain them by proposing a theoretical model.

In Table 1, *c*_g_ is the gas concentration; *T* is the operating temperature; *S* is the response; CNT is the carbon nanotubes; QDs is the quantum dots; IGZO is the indium gallium zinc oxide; NSs is the nanospheres; NShs is the nanosheets; P3HT is the poly(3-hexylthiophene); NWs is the nanowires; and *RT* is the room temperature.

## 2. Materials and Methods

TiO_2_ thin films were fabricated by the plasma-enhanced ALD technique using FlexAL ALD equipment (Oxford Instruments, Abingdon, UK). Thermally oxidized silicon plates (SiO_2_/Si) were used as substrates. Tetrakis(dimethylamido)titanium(IV) (TDMAT) [(CH_3_)_2_N]_4_Ti (99.999%) (Sigma-Aldrich, St. Louis, MO, USA) was used as the metal precursor with carrier gas of Ar (99.999%) at a flow rate of 200 cm^3^/min. Oxygen inductively coupled plasma (ICP) was used as an oxidizer. The discharge was excited in an oxygen atmosphere (99.999%) by a generator with a frequency of 13.56 MHz and a power of up to 300 W. The PEALD pulse durations were set at 0.8 s for TDMAT injection, 3 s for Ar purge, 3 s for exposure to plasma discharge, and 2 s for Ar purge. The growth rate at a temperature of 300 °C was 0.09 nm/cycle. The thickness of the deposited TiO_2_ films was 30 nm. A SENTECH Senduro spectral ellipsometer was used to estimate the thickness and the growth rate of TiO_2_ films at measurements in the wavelength range of 320–1800 nm.

The as-deposited TiO_2_ thin films were annealed at a temperature of 800 °C in an Ar atmosphere at a pressure of 2 kPa for 30 min. The rates of heating from *RT* to 800 °C and cooling from 800 °C to *RT* were 4 °C/min. The heating and cooling of samples were in an Ar atmosphere at a pressure of 2 kPa.

X-ray diffraction (XRD) was performed to determine the phase composition of the thin films and the crystal lattice parameters. XRD spectra of films were measured in a 2θ scanning mode employing a CuK_a_ radiation operated at 45 kV and 40 mA. The X-ray source wavelength was 1.5406 Å. The microrelief of the film surface was studied by a Bruker Dimension Icon atomic force microscope (AFM). Cross-sectional images of the annealed samples were examined by a Jeol JEM 2100 PLUS transmission electron microscope (TEM) at an accelerating voltage of 200 kV in a bright field (BF) mode. The elemental composition of the films was determined by the BF-TEM mode by means of a JEOL EX-24261M1G5T energy dispersive X-ray spectroscopy (EDX) analyzer at a beam current of 1 nA.

**Figure 1:**
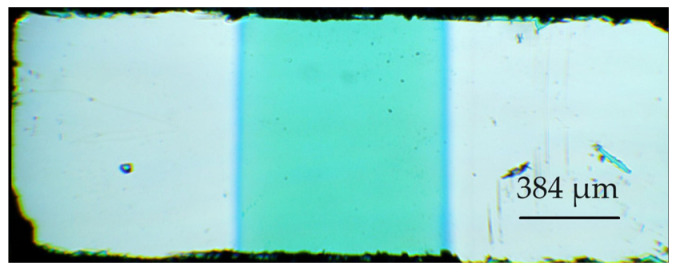
Microscopic photo of the sample based on TiO_2_ thin film.

To investigate the gas-sensitive properties, Pt contacts were deposited on the TiO_2_ film surface by means of vacuum deposition through a shadow mask. The plate with the film and contacts was divided into separate samples. The prepared samples were planar metal–semiconductor–metal (MSM) structures on SiO_2_/Si substrates (Figure 1). The interelectrode distance was kept at 1 mm. The thickness of the Pt contacts was about 330 nm.

The current–voltage (*I–V*) characteristics and time dependences of the sample’s resistance under the exposure to various gases were measured by means of a Keithley 2636A source meter and a sealed chamber with a Nextron MPS-CHH micro-probe station. A ceramic-type heater, installed in the sealed chamber, was used to heat the samples from *RT* to 700 °C with a temperature accuracy control of ±0.1 °C. The measurements were carried out under dark conditions and in a flow of dry N_2_, or in a gas mixture of dry N_2_ + dry O_2_. The flow rate of gas mixtures through the measurement chamber was maintained at 500 cm^3^/min. A pure dry air or a gas mixture of pure dry air + target gas was pumped through the chamber to examine the selectivity of the samples studied. H_2_, CO, CO_2_, NO_2_, NO and CH_4_ were selected as target gases. The source of pure dry air was a special generator. The concentration of the target gas in the mixture was controlled by a gas mixture generator with a Bronkhorst gas mass flow controller. The relative error of the gas flow rate did not exceed 1.5%. The voltage *U* applied to the samples during the measurements of time dependences of the resistance was kept at 3 V.

## 3. Results and Discussion

### 3.1. Structural Properties of the ALD-Deposited TiO_2_ Thin Films

**Figure 2:**
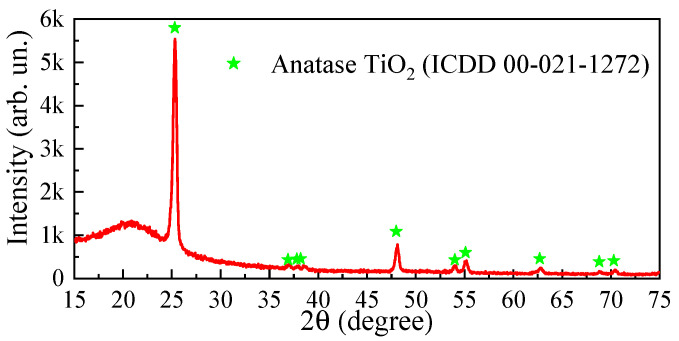
XRD patterns of the ALD-TiO_2_ thin films.

Figure 2 illustrates a typical XRD spectrum of the annealed ALD-TiO_2_ thin film. Several peaks appear at 2θ = 25.3°, 36.9°, 37.8°, 38.2°, 48.0°, 54.0°, 55.1°, 62.7°, 68.8° and 70.3°. These peaks are associated with (101), (103), (004), (112), (200), (105), (211), (204), (116) and (220) Bragg reflections of the tetragonal anatase TiO_2_ phase (ICDD 00-021-1272), respectively. These results confirm the polycrystalline nature of the material grown. The wide amorphous halo at 2θ ≈ 22° is due to the SiO_2_ layer. The parameters of the tetragonal crystalline lattice of the film are determined as *a* = 3.78 Å and *c* = 9.50 Å.

**Figure 3:**
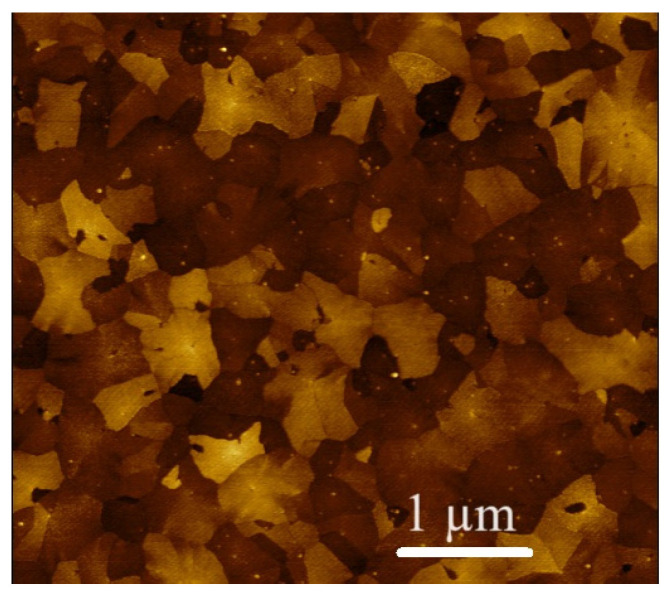
AFM images of annealed ALD-TiO_2_ thin film surface.

Figure 3 depicts typical annealed ALD-TiO_2_ film surface morphology images taken by AFM. The surface roughness parameters of the TiO_2_ thin films are *R*_a_ = 1.329 nm, *R*_q_ = 1.605 nm and *R*_z_ = 12.67 nm, where *R*_a_ is the arithmetic mean of the absolute values of the deviations of the film surface profile; *R*_q_ is the mean square value of the deviations of the film surface profile and *R*_z_ is the arithmetic mean of the greatest height of the profile of the film surface. *R*_q_ is lower than the value reported in ref. [21] devoted to ALD-deposited TiO_2_ thin films. The relatively high surface roughness should lead to an increase in the surface-to-volume ratio and the surface density of adsorption centers for gas molecules, and, as a result of this, to an increase in responses to gases [35].

**Figure 4:**
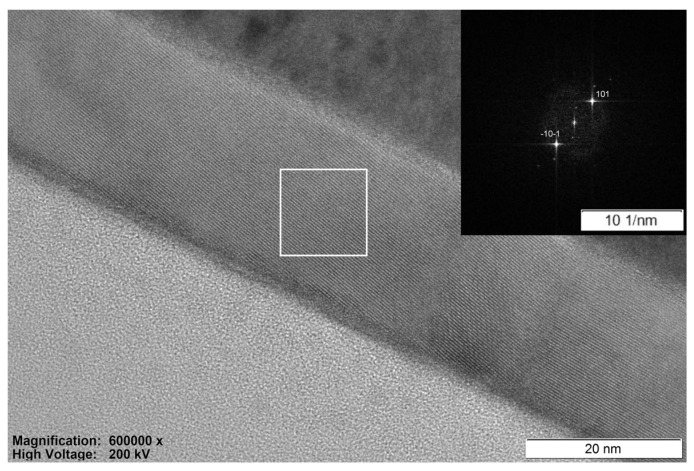
BF-TEM cross-sectional image of annealed ALD-TiO_2_ thin film on SiO_2_/Si substrate, the insertion is the diffraction pattern.

Figure 4 illustrates a BF-TEM cross-sectional image of annealed ALD-TiO_2_ thin film on a substrate in the high-resolution mode. The interplane distance *D* corresponding to the (101) reflection of the anatase TiO_2_ phase is 0.353 nm, determined by fast Fourier transformation (FFT). The *D* values for the same plane determined by FFT and by analysis of the XRD pattern (0.352 nm) are the same. TiO_2_ thin films have a nanocrystalline structure with amorphous inclusions according to the TEM study.

**Figure 5:**
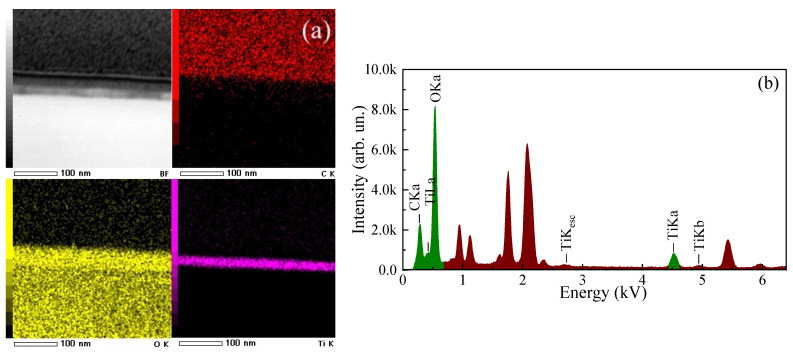
(**a**) Elementwise TEM-EDX mapping of cross-section of annealed ALD-TiO_2_ thin film; (**b**) EDX spectrum of annealed ALD-TiO_2_ thin film.

The contents of Ti and O elements in the films are measured to be ~27 at. % and ~73 at. % (Figure 5a,b), respectively. The increased O content in the films may be associated with features of the ALD process. There is also a peak corresponding to C in the EDX spectrum caused by the technological operations before the measurements of the spectrum.

### 3.2. Gas-Sensetive Properties of the ALD-Deposited TiO_2_ Thin Films

**Figure 6:**
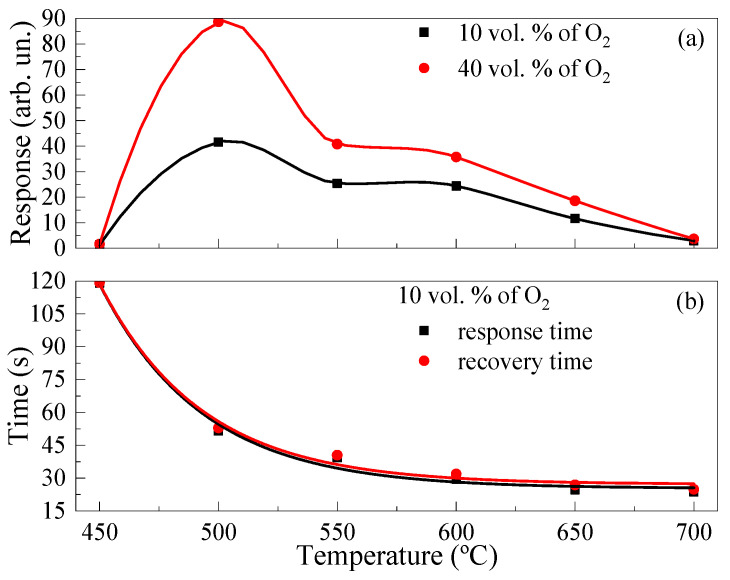
(**a**) Temperature dependences of response to 10 vol. % and 40 vol. % of O_2_; (**b**) Temperature dependences of response and recovery times upon exposure to 10 vol. % of O_2_.

At the next stage, the gas-sensitive properties of the ALD synthesized TiO_2_ thin films were investigated in detail. The exposure to O_2_ led to a reversible increase in the resistance of TiO_2_ thin films. The following ratio was chosen as the response *S*_ox_ of samples to O_2_:

*S*_ox_ = *R*_ox_/*R*_N_, (1)

where *R*_ox_ is the resistance of TiO_2_ thin film in a gas mixture of dry N_2_ + dry O_2_; *R*_N_ is the resistance of TiO_2_ thin film in dry N_2_ atmosphere. The temperature dependencies of the responses under the exposure to 10 vol. % and 40 vol. % of O_2_ (Figure 6a) had a maximum at *T* = 500 °C.

The samples practically did not react when exposed to O_2_ and had a high resistance, making it impossible to reliably register the response to gases at *T* < 450 °C. The presence of a maximum on the temperature dependence of the response is due to the influence of temperature on the processes of dissociation, adsorption/desorption of O_2_ molecules, and is specific to metal oxide semiconductors [36,37]. The response *t*_res_ and recovery *t*_rec_ times were determined upon exposure to 10 vol. % of O_2_ according to the method described in ref. [38] to estimate the operation speed of the films studied. The obtained values of *t*_res_ and *t*_rec_ can only be used to compare the operation speed of sensors under similar experimental conditions. We note that the *t*_res_ and *t*_rec_ decrease exponentially with the increase in *T* (see Figure 6b). It is also worth noting that *t*_res_ and *t*_rec_ were practically the same at *T* = 450–700 °C. The *t*_res_ and *t*_rec_ did not exceed 30 s in the range of *T* = 600–700 °C. The *t*_res_ and *t*_rec_ were 51.5 s and 52.9 s, respectively, at temperature of the maximum response to O_2_.

**Figure 7:**
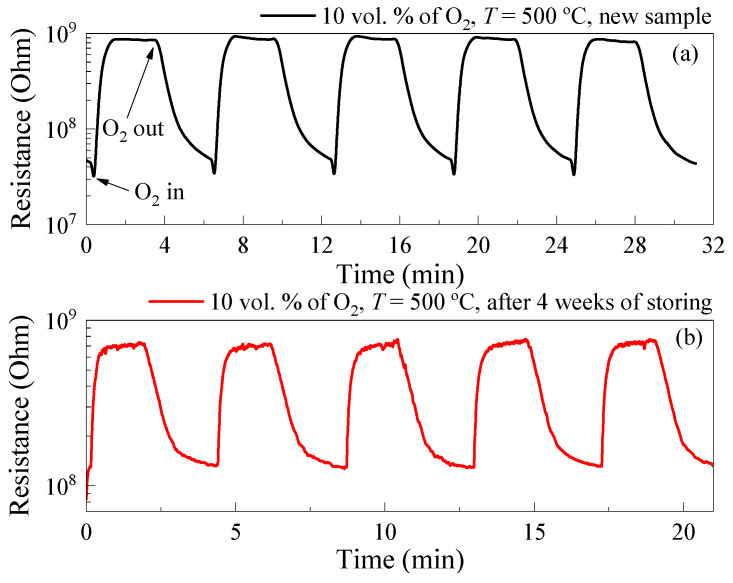
Time dependence of resistance upon cyclic exposure to 10 vol. % of O_2_ at *T* = 500 °C for new sample (**a**) and after 4 weeks of storing (**b**).

*R*_ox_ and *R*_N_ decreased by 4% and 36%, correspondingly, during a cyclic exposure to 10 vol. % of O_2_ (five cycles) (illustrated in Figure 7a); as a result, *S*_ox_ increased by 50%. On the other hand, the response of the films to O_2_ decreased by 6–7 times during storing in a sealed box at *RT* after the experiments at high *T* (Figure 7b) mainly due to a significant increase in *R*_N_. The healing of oxygen vacancies in TiO_2_ may be the reason for the increase in *R*_N_ at high *T* and exposure to high O_2_ concentrations [39]. This process is inertial and, consequently, manifested during prolonged testing of samples. To further stabilize the gas-sensitive properties of the films, doping with metal additives should be applied [40].

The rise of resistance *R* of the TiO_2_ thin film under the exposure to O_2_ and the drop of resistance after this exposure were approximated by the following functions, respectively:

*R*(*t*) = *R*^st^_ox_ − *A* × exp[−*t*/τ_1_], (2)

*R*(*t*) = *R*^st^_N_ + *B* × exp[−*t*/τ_2_], (3)

where *t* is time; *R*^st^_ox_ is the stationary resistance of TiO_2_ thin films in a gas mixture of dry N_2_ + dry O_2_; *R*^st^_N_ is the stationary resistance of TiO_2_ thin films in dry N_2_ atmosphere; *A* and *B* are constants; τ_1_ and τ_2_ are time constants. τ_1_ ≈ 23 s and τ_2_ ≈ 25 s at *T* = 500 °C and exposure to 10 vol. % of O_2_ for new samples, τ_1_ ≈ 13 s and τ_2_ ≈ 23 s for samples after 4 weeks of storing. The time constants τ_1_ and τ_2_ are related to the relaxation times of adsorption and desorption of oxygen molecules on the semiconductor surface.

**Figure 8:**
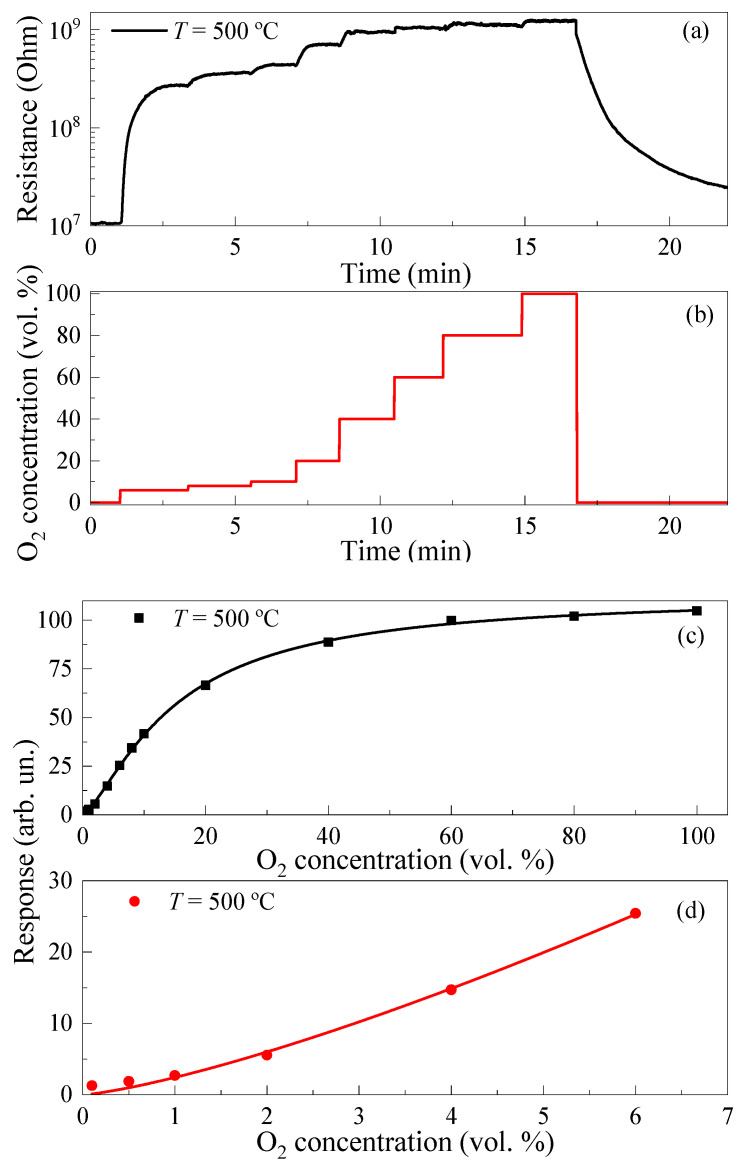
(**a**) Time dependence of resistance under stepwise increase in the O_2_ concentration at *T* = 500 °C; (**b**) Time profile of O_2_ concentration changes; (**c**) dependence of response on O_2_ concentration in dynamic range from 0.1 vol. % to 100 vol. % of O_2_ at *T* = 500 °C; (**d**) dependence of response on O_2_ concentration in dynamic range from 0.1 vol. % to 6 vol. % of O_2_ at *T* = 500 °C.

Figure 8a illustrates the time dependence of resistance of TiO_2_ thin films at *T* = 500 °C and stepwise increase in the O_2_ concentration *c*_ox_ (Figure 8b). The dependences of the response of TiO_2_ thin films on *c*_ox_ at *T* = 500 °C in dynamic range from 0.1 vol. % to 100 vol. % of O_2_ and 0.1 vol. % to 6 vol. % of O_2_ are presented in Figure 8c,d, correspondingly. The samples demonstrate a wide dynamic range from 0.1 vol. % to 100 vol. % of O_2_, but their responses to *c*_ox_ < 1 vol. % are low. Detailed research is needed to enhance oxygen sensitivity at these low oxygen concentration ranges.

**Figure 9:**
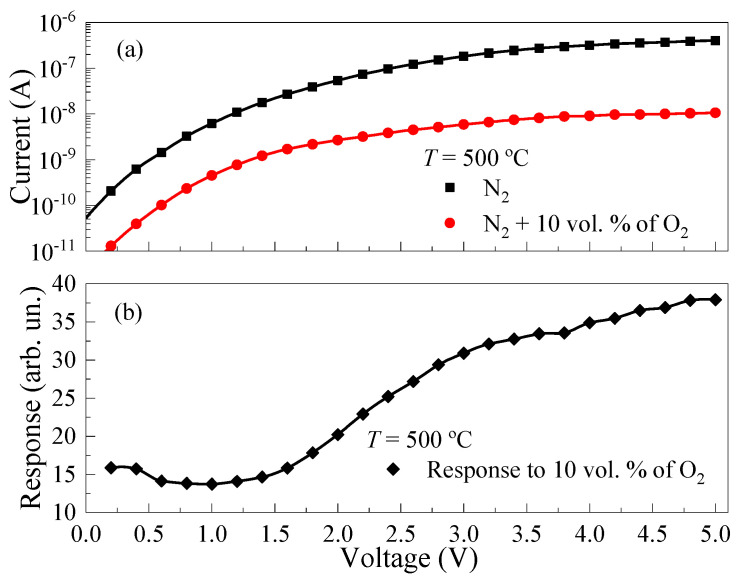
(**a**) *I*–*V* characteristics in N_2_ atmosphere and gas mixture of N_2_ + 10 vol. of O_2_ at *T* = 500 °C; (**b**) dependencies of the responses to 10 vol. % of O_2_ on applied voltage at *T* = 500 °C.

The effect of applied voltage on the response of TiO_2_ thin films to O_2_ was evaluated. The *I*–*V* characteristics of the samples were measured in dry N_2_ atmosphere and in a dry gas mixture of N_2_ + 10 vol. % of O_2_ (shown in Figure 9a). The *I*–*V* characteristics were approximated by the power function *I*~*U^z^*, where *I* is the electric current; *z* is a power index. The *z* value was 2.62 ± 0.05 in the N_2_ atmosphere and 2.22 ± 0.05 in the gas mixture of N_2_ + 10 vol. % of O_2_. The nonlinearity of the *I*–*V* characteristics was probably caused by the manifestation of an energy barrier at the Pt/TiO_2_ interface. The response of TiO_2_ thin films to O_2_ in the range of *U* = 0.2–1.5 V practically did not change with voltage (see Figure 9b). *S*_ox_ increased according to the power law *S*_ox_~*U^k^* with the increase in *U* from 1.5 V to 5 V, where *k* is a power index. *k* was 0.62 ± 0.05 at *c*_ox_ = 10 vol. % and at *T* = 500 °C.

**Figure 10:**
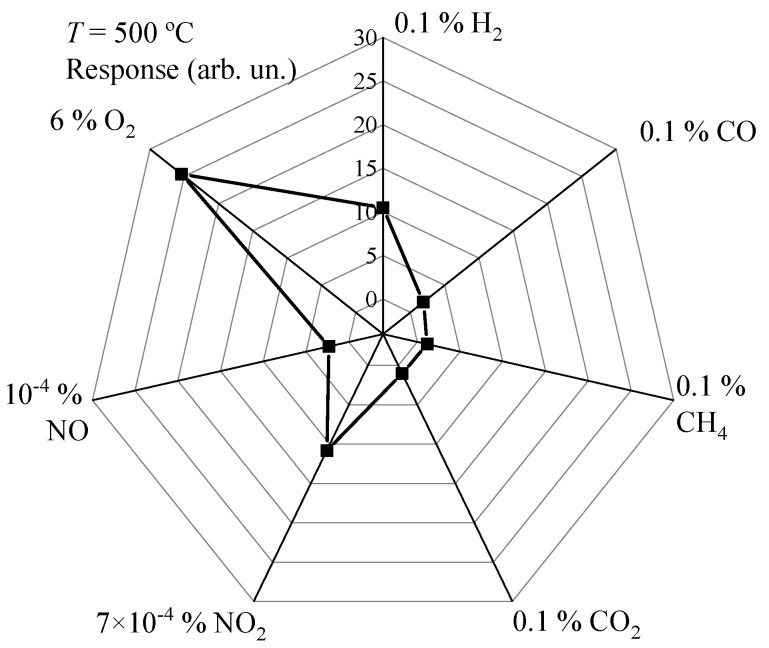
Responses to fixed concentrations of O_2_, H_2_, CO, CH_4_, CO_2_, NO_2_ and NO at *T* = 500 °C.

A promising application of O_2_ sensors based on the ALD-TiO_2_ thin films is the monitoring of the exhaust gases of the internal combustion engines. In order to achieve this, it is necessary to measure the change in O_2_ concentration in the range of 6–10 vol. % in the exhaust gas mixture [2,41]. In addition to O_2_, exhaust gases contain relatively high concentrations of H_2_, NO_x_, CH_x_, CO and CO_2_ [2,41,42]. To create a gas mixture corresponding to exhaust gas is difficult. But the sensitivity of the ALD-TiO_2_ thin films to these gases with concentrations close to those of exhaust gases was investigated at the temperature of the maximum response to O_2_. The ALD-TiO_2_ thin films demonstrated a relatively high response to H_2_, NO and NO_2_. The experimental results are exhibited in Figure 10. The responses to relatively high concentrations of CO, CH_4_ and CO_2_ were insignificant or absent. Exposure to H_2_ led to a drop in the resistance of the films. The ratio of resistances in the pure dry air and in the gas mixture of pure dry air + reducing gas (H_2_, CO and CH_4_) was chosen as response. The exposure to NO and NO_2_ led to an increase in the TiO_2_ thin film resistance. The response to these gases was determined as a ratio of the resistances in the gas mixture of pure dry air + NO (NO_2_) and in the pure dry air. It is worth noting that the responses to 0.1 vol. % of H_2_ and 7 × 10^−4^ vol. % of NO_2_ were the same. This indicates the high sensitivity of the films to low NO_2_ concentrations.

### 3.3. The Mechanism of the Sensory Effect

The ALD-deposited TiO_2_ thin films annealed at *T*_ann_ = 800 °C in Ar for 30 min are corresponding to the anatase phase. They are homogeneous and relatively smooth, without features of microrelief on the film surface which could affect the transport of charge carriers through the film. Therefore, the increase in the resistance of such TiO_2_ films under exposure to oxygen is due to the chemisorption of O_2_ molecules on their surface. During chemisorption, oxygen captures electrons from the TiO_2_ conduction band and forms a region depleted by charge carriers in the near-surface part of the semiconductor film, with a width *W*. There are no charge carriers in this region, and the electric current between the contacts flows through a layer of thickness (*d* − *W*), which is called a conduction channel. The negative charge on the surface of the *n*-type semiconductor film leads to the formation of the upward bending of energy band *eV*_s_, where vs. is the surface potential; *e* is the electron charge. It is shown that *eV*_s_~*N_i_*^2^ [43,44], where *N_i_* is the surface density of chemisorbed oxygen ions. The relationship between *W* and *eV*_s_ has the following form:

*W = L*_D_ × [2*eV*_s_/(*kT*)]^0.5^, (4)

where *L*_D_ = [(εε_0_*kT*/(*e*^2^*n*)]^0.5^; *k* is the Boltzmann constant; ε is the electric constant. The chemisorption of oxygen on the surface of TiO_2_ thin films leads to an increase in *W* and *eV*_s_, as well as to a decrease in the thickness of the conduction channel that leads to an increase in the resistance of the film. At *n* ≈ 10^18^ cm^−3^, *L*_D_ for the anatase TiO_2_ phase increases linearly from 8.0 nm to 9.3 nm and does not exceed the film thickness. The TiO_2_ film resistance in gas mixture N_2_ + O_2_ is given by

R_O_ = *ρ*_N_*l*[*b*(*d* − *W*)], (5)

where *ρ*_N_ is the resistivity of the TiO_2_ film in the dry N_2_ atmosphere; *l* is the distance between the electrodes; *b* is the width of the TiO_2_ film. The intrinsic surface states can be neglected for ionic semiconductors [43]. Thus, in the N_2_ atmosphere, *eV*_s_ and *W* = 0, and *R*_N_ = *ρ*_N_*l*/(*bd*). The expression for response to oxygen is

*S*_ox_ = (1 *− W/d*)^−1^. (6)

**Figure 11:**
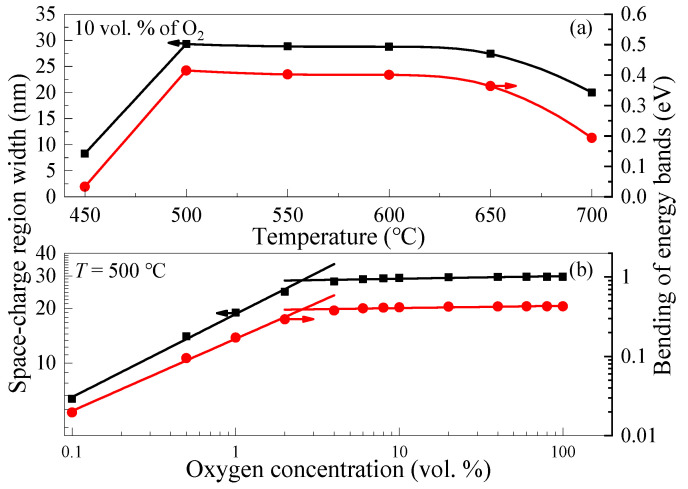
(**a**) Dependences of space-charge region width and bending of energy bands on temperature at *c*_ox_ = 10 vol. % of O_2_; (**b**) dependences of space-charge region width and bending of energy bands on oxygen concentration at *T* = 500 °C.

High *S*_ox_ takes place when the region depleted by charge carriers extends almost the entire thickness of the film, but there is a very thin conduction channel. The dependences of *W* and *eV*_s_ on *T* and *c*_ox_ (Figure 11a,b) are estimated by means of experimental *S*_ox_ and calculated *L*_D_ values. Pure TiO_2_ thin films do not demonstrate reliably recorded sensitivity to O_2_ at *T* < 450 °C. An increase in *T* stimulates dissociative adsorption of O_2_ molecules. At the same time, high *W* and *eV*_s_ are observed in the range of *T* = 500–650 °C, indicating a high surface density of chemisorbed O^−^ ions. A further increase in *T* leads to a predominance of O^−^ desorption, which leads to a sharp decrease in *W* and *eV*_s_, as well as the response of films. There are two linear areas on the dependencies of *W* and *eV*_s_ on the O_2_ concentration in double logarithmic coordinates (Figure 11b). *eV*_s_~*c*_ox_^*l*_1_^ and *W*~*c*_ox_^*m*_1_^ in the range of *c*_ox_ = 0.1–2 vol. %, where *l*_1_ and *m*_1_ are the power indexes. *l*_1_ = 0.90 ± 0.03 and *m*_1_ = 0.45 ± 0.01. The equality *m*_1_ = *l*_1_/2 follows from Expression (4). There are weaker power dependencies of *eV*_s_~*c*_ox_^*l*_2_^ and *W*~*c*_ox_^*m*_2_^ in the range of *c*_ox_ = 4–100 vol. %, where *l*_2_ and *m*_2_ are the power indexes. At the same time, *l*_2_ = 0.028 ± 0.006 and *m*_2_ = 0.014 ± 0.003. We assume that the manifestation of two linear areas on the dependencies is due to the presence of two types of adsorption centers for oxygen molecules. The possibility of this was shown for SnO_2_ and Ga_2_O_3_ thin films [45,46]. As a result, oxygen is chemisorbed in the form of O^2−^ on the first type of adsorption centers and in the form of O^−^ on the second one. Chemisorption on the centers of the first type prevails in the range of *c*_ox_ = 0.1–2 vol. %. Twice as many electrons are captured during the chemisorption of one oxygen molecule on this type of centers, which causes a sharp increase in *W* and *eV*_s_. Centers of the second type prevail in the range of *c*_ox_ = 4–100 vol. % and the dependencies of *W* and *eV*_s_ on *c*_ox_ are much weaker. In this case, *eV*_s_~(*N_i_*_1_ + *N_i_*_2_)^2^, where *N_i_*_1_ is the surface density of chemisorbed O^2−^ ions; *N_i_*_2_ is the surface density of chemisorbed O^−^ ions. It is worth noting that it is possible to approximate the experimental dependence of the response on the O_2_ concentration (see Figure 8c) by means of Expressions (4) and (6) in the case when *eV*_s_(*c*_ox_) = *a*(*c*_ox_) × *c*_ox_^*l*_1,2_^, where *a* is a function of *c*_ox_. We believe that this dependence of *eV*_s_(*c*_ox_) is due to the manifestation of the dependence of the surface density of adsorption centers for oxygen molecules on *c*_ox_.

The effect of applied voltage on the response of metal oxide to gases has been poorly studied in the literature so far. For SnO_2_ films, it is shown that negatively charged ions of chemisorbed oxygen diffuse over the surface, participating in the transport of the electric current [47]. The diffusion time of adsorbed oxygen over the surface is less than its lifetime on the surface at high electric fields. Negatively charged ions accumulate near the anode and form a high-resistance region at higher electric fields. An additional increase in the resistance contributes to an increase in response at higher *U*.

Within the framework of the proposed mechanism of the sensory effect, the sensitivity of the films to reducing gases is due to the interaction of their molecules with previously chemisorbed oxygen, as a result of which *W* and *eV*_s_, as well as the resistance of TiO_2_ films, decrease. The mechanism of sensitivity of TiO_2_ films to reducing gases was previously described in detail by our group in ref. [48]. Oxidizing gases interact with the surface of TiO_2_ films like oxygen.

The proposed sensory effect does not take into account the contribution of changes in the potential barrier at the Pt/TiO_2_ interface under the exposure to gases. The Schottky barrier between a semiconductor and catalytically active metals such as Pt, Pd, Ru, Ir, and Ag, including Pt/TiO_2_ structures, are known to exhibit sensitivity to H_2_ and other gases [38,49,50]. According to the corresponding sensory effect’s mechanism, gas molecules (H_2_ for example) undergo a dissociative adsorption on the catalytically active metal surface. Then, H atoms diffuse through the metal layer to the metal–semiconductor interface. A dipole layer of H atoms is formed at this interface, which leads to a decrease in the height of a potential barrier for electrons at the metal–semiconductor interface and an increase in the current. The diffusion of H atoms in Pt is characterized by lower diffusion activation energies and higher diffusion coefficients than those for the diffusion of O atoms in Pt [51,52,53,54,55,56]. It can be estimated that the diffusion times of H atoms through a 330 nm thick Pt layer are less than 0.055 s. In contrast, the diffusion time of O atoms through the Pt contact layer is about 10^8^ s. Thus, changes in the potential barrier at the Pt/TiO_2_ interface under the exposure to O_2_ should not be taken into account. However, this effect may be the main one under the exposure to other gases, such as H_2_ and CO.

The gas-sensitive characteristics of TiO_2_ films synthesized by different deposition methods under the exposure to O_2_ are compared in Table 2, where NRA is nanorod array; NPs is the nanoparticles; NFs are the nanoflakelets; NTs is the nanotubes; RFMS is the radio frequency magnetron sputtering; DCMS is the direct current magnetron sputtering; IBSD is the ion beam sputtering deposition; GLAD + EBE is the glancing angle deposition with electron beam evaporation; AVO is the acid vapor oxidation; USP is the ultrasonic spray pyrolysis; TD + HM is the thermal decomposition assisted hydrothermal method; PEO is the plasma electrolytic oxidation; AO is the anodic oxidation; UV is the exposure to ultraviolet radiation. The gas-sensitive properties of ALD-deposited undoped TiO_2_ thin films studied in this work are comparable or superior to the results reported for undoped TiO_2_ thin films grown by other methods. High *S*_ox_ requires heating of the structures to *T* > 300 °C. *T* is reduced to *RT* by using of the ultrathin films or nanostructures, exposure to UV. However, at the same time, *t*_res_ and *t*_rec_ significantly increase. On the other hand, an increase in *S*_ox_ is achieved by doping of TiO_2_ with Nb, Pd and Cr, introducing ZrO_2_, MoO_3_, forming of multilayer structures, and modifying the surface of films with nanoparticles.

## 4. Conclusions

The structural and gas-sensitive properties under the exposure to O_2_ within the temperature range from 30 °C to 700 °C of TiO_2_ thin films deposited by atomic layer deposition on SiO_2_/Si substrates were studied. The structure of the films annealed at 800 °C in an Ar for 30 min corresponded to the anatase phase. They are homogeneous and relatively smooth. The ALD-TiO_2_ thin films demonstrated high responses to O_2_ in the dynamic range from 0.1 to 100 vol. % and to low concentrations of H_2_, NO_2_. The greatest response—41.5 arb. un.—was observed at a temperature of 500 °C under exposure to 10 vol. % of O_2_. A mechanism describing the sensory effect in the ALD-TiO_2_ thin films was proposed. The resistance of the films increases due to the chemisorption of oxygen molecules on their surface that decreases the thickness of the conduction channel between the metal contacts. It was suggested that there are two types of adsorption centers on the TiO_2_ thin films surface: oxygen is chemisorbed in the form of O^2–^ on the first one and O^–^ on the second one.

## Figures and Tables

**Table 1:** Comparison of gas-sensitive characteristics of ALD-deposited metal oxide thin films.

Material	*d* (nm)	Gas	*c*_g_ (ppm)	*T* (°C)	*S* (arb. un.)	Ref.
TiO_2_	50	NH_3_	100	350	4.24	[21]
CNT/TiO_2_	10 (TiO_2_)	NO_2_	8	150	~10	[23]
SnO_2_	17.5	C_2_H_5_OH	500	300	1.64	[24]
SnO_2_	10	CO	10^4^	450	~21	[25]
WO_3_	6.5	C_2_H_5_OH	100	275	~14	[26]
Ga_2_O_3_	1.5				~1.4	
Ga_2_O_3_/WO_3_	1.5/6.5				~3.5	
SnO_2_	90	H_2_	1000	400	~380	[27]
TiO_2_/SnO_2_ QDs	30 (TiO_2_)	CO	1	300	1.8	[28]
SnO_2_	4.4	C_2_H_5_OH	200	400	20	[29]
In_2_O_3_/SnO_2_	4/4.4			350	37	
IGZO	150	NO_2_	100	200	5154	[30]
ZnO/SnO_2_ NSs	24 (ZnO)	HCHO	20	200	38.2	[31]
Fe_2_O_3_/SnO_2_ NShs	20 cycles	HCHO	20	220	4.57	[32]
*p*-TiO_2_	70	NO	10	*RT*	1.244	[33]
P3HT/ZnO NWs	-	NH_3_	5	*RT*	1.35	[34]

**Table 2:** Comparison of sensitivity to O_2_ for TiO_2_ thin films deposited by different methods.

Material	Methods	*d* (nm)	*c*_ox_ (vol. %)	*T* (°C)	*S*_ox_ (arb. un.)	Ref.
TiO_2_	RFMS	50	0.6	500	1.14	[18]
TiO_2_	DCMS	60	10	*RT*	76	[5]
TiO_2_	IBSD	130	40	750	7.64	[48]
TiO_2_	sol-gel	-	2	700	6.5	[6]
Nb (6%):TiO_2_		-			73.2	
TiO_2_	sol-gel	-	1	400	4.4	[17]
TiO_2_ + ZrO_2_ (10 mol. %)		-			5	
TiO_2_	RFMS	30	10	*RT* + UV	~5.5	[4]
Cr-TiO_2_/TiO_2_	GLAD + EBE	-			~9	
TiO_2_ NRA	AVO	-	8	*RT*	~1.9	[20]
TiO_2_	USP	-	0.1	300	5	[57]
TiO_2_-Ag NPs		-			9	
Pd:TiO_2_	sol-gel	~110	1→20	240	1.27	[19]
TiO_2_	sol-gel	-	0.1	420	28	[58]
TiO_2_ + MoO_3_ (25 at. %)				370	30	
Au (6 nm)/TiO_2_	RFMS	300	5	400	61.3	[59]
VO_x_/TiO_2_ NFs	TD + HM	-	0.01	500	1.32	[60]
Pt/TiO_2_	PEO	-	10	*RT*	2	[61]
TiO_2_ NTs	AO	-	4	100	160	[62]
TiO_2_	sol-gel	-	4	252	1.16	[63]
TiO_2_	ALD	23	0.1	500	1.27	This work
			1		2.70	
			2		5.55	
			10		41.61	

## Data Availability

Not applicable.
